# Vitamin D Supplementation and Disease-Free Survival in Stage II Melanoma: A Randomized Placebo Controlled Trial

**DOI:** 10.3390/nu13061931

**Published:** 2021-06-04

**Authors:** Harriet Johansson, Giuseppe Spadola, Giulio Tosti, Mario Mandalà, Alessandro M. Minisini, Paola Queirolo, Valentina Aristarco, Federica Baldini, Emilia Cocorocchio, Elena Albertazzi, Leonardo Zichichi, Saverio Cinieri, Costantino Jemos, Giovanni Mazzarol, Patrizia Gnagnarella, Debora Macis, Ines Tedeschi, Emanuela Omodeo Salè, Luigia Stefania Stucci, Bernardo Bonanni, Alessandro Testori, Elisabetta Pennacchioli, Pier Francesco Ferrucci, Sara Gandini, on behalf of the Italian Melanoma Intergroup (IMI)

**Affiliations:** 1 Division of Cancer Prevention and Genetics, IEO, European Institute of Oncology IRCCS, 20141 Milan, Italy; valentina.aristarco@ieo.it (V.A.); debora.macis@ieo.it (D.M.); bernardo.bonanni@ieo.it (B.B.); 2 Divisione di Chirurgia del Melanoma, IRCCS Fondazione Istituto Nazionale per lo Studio e la Cura dei Tumori, 20133 Milan, Italy; giuseppe.spadola@istitutotumori.mi.it; 3 Division of Surgery for Melanoma, Sarcoma, and Rare Tumors, IEO, European Institute of Oncology IRCCS, 20141 Milan, Italy; giulio.tosti@ieo.it (G.T.); federica.baldini@ieo.it (F.B.); ines.tedeschi@ieo.it (I.T.); elisabetta.pennacchioli@ieo.it (E.P.); 4 Unit of Medical Oncology, Department of Oncology and Haematology, Papa Giovanni XXIII Cancer Center Hospital, 24127 Bergamo, Italy; mario.mandala@unipg.it; 5 Department of Oncology, Azienda Sanitaria Universitaria del Friuli Centrale, 33100 Udine, Italy; alessandro.minisini@asuiud.sanita.fvg.it; 6 Department of Medical Oncology, IRCCS Ospedale Policlinico San Martino—IST-Istituto Nazionale per la Ricerca sul Cancro, 16132 Genoa, Italy; paola.queirolo@ieo.it; 7 Division of Medical Oncology for Melanoma, Sarcoma and Rare Tumors, IEO, European Institute of Oncology IRCCS, 20141 Milan, Italy; emilia.cocorocchio@ieo.it; 8 San Raffaele Telethon Institute for Gene Therapy, IRCCS San Raffaele Scientific Institute, 20132 Milan, Italy; albertazzi.elena@hsr.it; 9 Dermatology Unit, Ospedale S.A. Abate, 91016 Erice, Italy; l.zichichi@gmail.com; 10 Medical Oncology & Breast Unit, Department of Oncology, “Antonio Perrino” Hospital, 72100 Brindisi, Italy; saverio.cinieri@asl.brindisi.it; 11 Division of Pharmacy, IEO, European Institute of Oncology IRCCS, 20141 Milan, Italy; costantino.jemos@ieo.it (C.J.); eoomodeo@ieo.it (E.O.S.); 12 Division of Pathology, IEO, European Institute of Oncology IRCCS, 20141 Milan, Italy; giovanni.mazzarol@ieo.it; 13 Division of Epidemiology and Biostatistics, IEO European Institute of Oncology IRCCS, 20141 Milan, Italy; patrizia.gnagnarella@ieo.it; 14 Section of Medical Oncology, Department of Biomedical Sciences and Clinical Oncology (DIMO), University of Bari ‘Aldo Moro’, 70124 Bari, Italy; stuccistefania@gmail.com; 15 EORTC Melanoma Group, 1200 Brussel, Belgium; info@alessandrotestori.it; 16 Skin Oncology Division, Image Rigenerative Clinic, 20121 Milan, Italy; 17 Biotherapy of Tumors Unit, IEO, Department of Experimental Oncology, European Institute of Oncology, IRCCS, 20141 Milan, Italy; pier.ferrucci@ieo.it; 18 Department of Experimental Oncology, IEO, European Institute of Oncology IRCCS, 20141 Milan, Italy; sara.gandini@ieo.it

**Keywords:** vitamin D, melanoma, cancer, Breslow, prognosis, single-nucleotide polymorphisms

## Abstract

Patients with newly resected stage II melanoma (*n* = 104) were randomized to receive adjuvant vitamin D3 (100,000 IU every 50 days) or placebo for 3 years to investigate vitamin D3 protective effects on developing a recurrent disease. Median age at diagnosis was 50 years, and 43% of the patients were female. Median serum 25-hydroxy vitamin D (25OHD) level at baseline was 18 ng/mL, interquartile range (IQ) was 13–24 ng/mL, and 80% of the patients had insufficient vitamin D levels. We observed pronounced increases in 25OHD levels after 4 months in the active arm (median 32.9 ng/mL; IQ range 25.9–38.4) against placebo (median 19.05 ng/mL; IQ range 13.0–25.9), constantly rising during treatment. Remarkably, patients with low Breslow score (<3 mm) had a double increase in 25OHD levels from baseline, whereas patients with Breslow score ≥3 mm had a significantly lower increase over time. After 12 months, subjects with low 25OHD levels and Breslow score ≥3 mm had shorter disease-free survival (*p* = 0.02) compared to those with Breslow score <3 mm and/or high levels of 25OHD. Adjusting for age and treatment arm, the hazard ratio for relapse was 4.81 (95% CI: 1.44–16.09, *p* = 0.011). Despite the evidence of a role of 25OHD in melanoma prognosis, larger trials with vitamin D supplementation involving subjects with melanoma are needed.

## 1. Introduction

Cutaneous malignant melanoma is a major public health problem in many countries. In recent decades, its incidence has increased faster than that of all other cancers, although fortunately this increase has not been followed by a similar rise in mortality rates, possibly reflecting the positive impact of education and prevention programs [1]. In Italy, the age-standardized incidence rate reported in 2020 was 12.6 per 100,000 person per year in males and 12.0 in females, with mortality rates around 1.5 (https://gco.iarc.fr/today/data/factsheets/populations/380-italy-fact-sheets.pdf (accessed on March 2021)).

Family history of melanoma, some germline mutations, multiple benign or atypical nevi, and previous diagnosis of melanoma are major risk factors for melanoma development. Breslow thickness is the single most important prognostic factor for clinically localized primary melanoma [2]. It defines the tumor depth from the surface of the epidermal granular layer to the point of maximum tumor thickness.

Low phototype and exposure to ultraviolet light are important risk factors for melanoma development. It is well known that sunburns and intermittent sun exposure increase the risk for melanoma, while a continuous pattern of sun exposure, in contrast, appears to be inversely associated with melanoma [3], plausibly through cutaneous vitamin D synthesis. Orally ingested vitamin D rapidly attenuates inflammation following sunburn [4], suggesting an anti-inflammatory role in skin homeostasis.

Skin is a key source of vitamin D in humans. A precursor in the epidermis is photo-activated by ultraviolet B radiation into provitamin D3 (cholecalciferol). Through a series of modifications in the skin, liver, and kidneys the vitamin D precursors are metabolized into the physiologically active metabolite 1,25 dihydroxyvitamin D (calcitriol). The intermediate metabolite 25-hydroxyvitamin D is the most abundant in blood and considered the best indicator of individual’s vitamin D status because of its long half-life. Although there is still some discussion on the matter, vitamin D insufficiency is most commonly defined by serum levels of 25OHD between 21 and 29 ng/mL, and vitamin D deficiency is defined as levels of 25OHD lower than 20 ng/mL [5].

Apart from its central role in skeletal health, growing evidence suggests vitamin D plays an important role in a wide range of diseases including inflammatory disease, immune response, cardiovascular health, and carcinogenesis, with different levels of evidence [6,7,8]. Studies in vitro and in vivo demonstrate that calcitriol exerts pleiotropic anticancer effects via binding to its vitamin D Receptor (VDR). It regulates the expression of tumor-related genes and mediates inhibition of cell growth, adhesion, migration, metastases and angiogenesis [9]. Such effects have also been confirmed specifically for malignant melanoma [10].

Case–control studies have shown higher serum 25OHD levels in healthy controls than in melanoma patients at the time of diagnosis. A multivariate model suggested that both deficient and insufficient serum levels of vitamin D are associated with melanoma and revealed a trend to reduced risk of melanoma when vitamin D approaches its normal values [11].

The hypothesis that higher levels of vitamin D reduce cancer incidence or mortality has been tested through randomized controlled trials that provide high-level evidence. A recent meta-analysis including 10 randomized clinical trials that attained circulating 25OHD concentrations of 54–135 nmol/L in the intervention group [12] found that vitamin D supplementation significantly reduced total cancer mortality, but not total cancer incidence. Most of the studies were not initially designed to test the hypothesis that vitamin D affects the risk for cancer occurrence.

These data further support previously published studies reporting a correlation of serum vitamin D levels with clinical outcome of melanoma patients, including a relationship between lower Breslow tumor thickness and high 25OHD levels [13].

A prospective cohort study of 1042 melanoma patients [14] found a significant association of low 25OHD levels with worse melanoma prognostic factors such as high tumor thickness, ulcerated tumor, advanced melanoma stage, and increased levels of C-reactive protein (CRP), an important biomarker of inflammation and cardiovascular risk [15].

We designed a multicenter double-blind placebo-controlled adjuvant trial to assess the effect of a 3-year vitamin D supplementation (100,000 IU/every 50 days) on disease-free survival of melanoma patients in stage II.

## 2. Materials and Methods

### 2.1. Study Participants

Study participants were 104 adults aged 75 years or younger with recent resected stage II cutaneous malignant melanoma. Participants had to have hematopoietic, hepatic, and renal functionality within normal ranges. Main exclusion criteria were current use of at least 600 IU/day of supplemental vitamin D or high-dose calcium therapy within the prior 6 month and any prior cancer or other significant diseases that could hamper the participant’s safety or preclude the benefit of vitamin D supplementation.

Potential study participants were recruited among patients treated at six Italian Institutes (Table S1). Routine laboratory tests on peripheral blood to check eligibility were performed at each center. A questionnaire to collect information on clinical features and recognized melanoma risk factors was applied [16,17,18].

All subjects gave informed consent before participating in the study, which was conducted in accordance with the Declaration of Helsinki. The study (N. EudraCT 2009-012049-46) was approved by the Ethics Committee of the European Institute of Oncology, Milan, the coordinating center, and following approval by the local ethics committees of all participating sites.

### 2.2. Intervention and Study Procedures

Study participants were randomized to receive an oral solution in ampoule every 50 days, containing 100,000 IU of vitamin D3 (an average of 2000 IU/day) or placebo administered for 3 years. Randomization blocks of 1:1 to one of the two treatment arms was achieved in a masked fashion by the Data Center at the European Institute of Oncology. Only the system administrator had access to the allocation sequences. Stratification criteria were center, age, ulceration, and Breslow thickness.

Drug supply was distributed by the pharmacy of the European Institute of Oncology. Both participants and clinicians were blinded as to the treatment arm. Pharmacists were the only un-blinded personnel in the trial staff. A calendar was given to the patient as a reminder of the dates for taking the ampoules. Adherence was also evaluated by measuring serum concentrations of 25OHD as a surrogate biomarker of compliance. Study participants received an 8 months’ supply of medication.

The participants were asked to return to the recruitment clinic for 13 follow-up visits at the 4th, 8th, 12th, 16th, 20th, 24th, 28th, 32nd, 36th, 42nd, 48th, 54th, and 60th month.

Adverse events were assessed using the NCI Common Terminology Criteria for Adverse events version 3.0, Wiley-Liss, New York, NY, USA, for toxicity and adverse event reporting.

### 2.3. Objectives

The main endpoint of the study was to assess the effect of vitamin D supplementation on recurrence in resected, stage II melanoma patients (disease-free survival).

Secondary endpoints were to investigate the association of changes in time of serum 25OHD levels and percentage of patients that reached the 25OHD cut-off level of sufficiency (>30 ng/mL from August to November and >20 ng/mL from January to July) during the 1st year, with melanoma outcome, and to assess the relationship between genetic polymorphisms (VDR and other genes involved in vitamin D metabolism), risk biomarkers (serum and histology), and melanoma prognosis (disease-free survival).

### 2.4. Laboratory Methods

The Laboratory at the Coordinating Center (IEO Lab) coordinated specimen collection and labelling for storage of samples for biomarker assessments and organized the reciprocal pick-up.

Whole EDTA-treated blood and serum samples for biomarker measurements were collected at baseline and at the 4th, 8th, 12th, 24 h, 36th, 48th, and 60th monthly visits (or early termination visit) and stored at −80 °C until biomarker measurement. Routine lab measurements at baseline for eligibility were performed locally. Amongst these, lactate dehydrogenase (LDH) and neutrophil–lymphocyte ratio (NLR) were included.

Serum 25OHD and PTH concentrations were determined in batches using commercially available competitive immune-chemiluminescent assays designed for the automated analyzer LIAISON^®^ (DiaSorin SpA, Saluggia, Italy). The 25OHD method recognizes both metabolites of vitamin D (D2-D3). C-reactive protein was measured with the Alinity high sensitivity C-reactive protein (hs-CRP) Vario assay, used for the quantitative immune-turbidimetric determination on the Alinity c analyzer (Abbott Park, IL, USA). The lower limit of detection is 0.04 mg/dL. Control samples provided with the assay kits as well as an in-house pooled serum control sample were run in each analytical session for monitoring intra- and inter-assay variability. When a CRP result was expressed as being <0.04 mg/dL samples were analyzed with a second method that has higher sensitivity, using the Atellica CH instrument (Siemens Healthineers, Erlangen, Germany) with a LoQ of <0.016 mg/dL.

Genomic DNA was extracted from peripheral blood specimens with the QIAamp DNA blood kit (Qiagen, Valencia, CA, USA), following the protocol of the manufacturer and using the automated platform “QIAcube”, and quantified using a NanoDrop spectrophotometer (Thermo Scientific, Wilmington, DE, USA). DNA samples were genotyped for a comprehensive set of single-nucleotide polymorphisms (SNPs). We analyzed Bsm1 (rs1544410), Taq1 (rs731236), Fok1 (rs228570), Apa1 (rs7975232) in the VDR gene; 3 SNPs involved in Vitamin D metabolism (CYP24A1-rs6013897, CYP27B1-rs10877012, CYP2R1-rs10741657), and 3 SNPs (rs2282679, rs7041 and rs4588) in the GC gene coding for the main transporter of Vitamin D in the circulation. SNPs were analyzed by the TaqMan SNP Genotyping Assays using the ABI PRISM 7500 FAST Real-Time PCR System (Thermo Fisher Scientific, Wilmington, DE, USA). Briefly, nearly 10 ng of DNA in 2 μL was added to a 10 μL reaction together with 8 μL of reaction mix containing forward and reverse primers and two allele-specific fluorescent labelled probes (one wild-type and one variant allele-specific). Control samples, representing a complete set of genotypes for all SNPs, were processed in each run. Hardy–Weinberg equilibrium (HW) for genotype frequencies was tested using a Chi-square test in the controls.

### 2.5. Statistical Methods

We investigated the association of vitamin 25OHD levels at baseline and after 1 year supplementation with disease-free survival by evaluating Kaplan–Meier curves and Log-rank. We categorized the 25OHD level accordingly with seasonal variability. We defined levels as insufficient or “low vitamin D” when 25OHD was below 20 ng/mL from December to July and below 30 ng/mL from August to November. The multivariable Cox proportional hazards model were adopted to assess the association of 25OHD levels with disease-free survival, taking into account the trial arms and other prognostic factors such as age and Breslow thickness. The association of 25OHD with relapse was assessed considering 25OHD at baseline and at 12 months, after one year of supplementation or placebo administration.

We also investigated the association of 25OHD at baseline and VDR polymorphisms with melanoma stage by univariate Chi-square test and multivariable logistic models. 

Random effects models for repeated measures were applied to investigate factors associated with changes in time of 25OHD, adjusting for baseline values. Normal distribution of residuals of the fully adjusted model was checked.

## 3. Results

**Figure 1:**
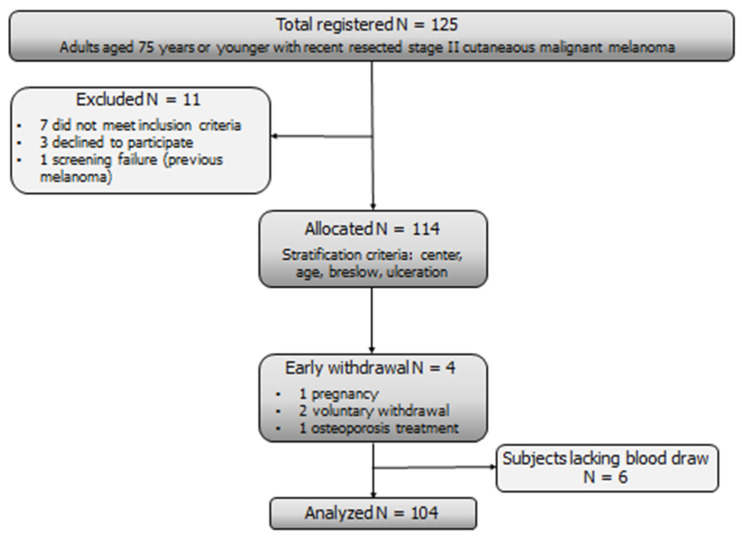
The participant flow diagram.

Between May 2011 and January 2017, 125 patients were enrolled in the study, and 114 were randomized. The participant flow diagram is shown in Figure 1: after excluding early withdrawal and participants who refused to give their blood for biomarker analysis, 104 subjects were eventually analyzed in this paper. The main participant and tumor characteristics were evenly distributed between the arms (Table 1). Median age at diagnosis was 51 years, 57% of the patients were male, and 43% were female. The majority of participants had low levels of 25OHD (80%), and we observed a trend to low 25OHD levels in patients with larger tumor size, although the difference was not significant (Table 2).

Median and interquartile ranges of serum 25OHD, PTH, and hs-CRP according to treatment arm and time interval are shown in Table 3. Serum levels of 25OHD sharply rose already after 4 months of supplementation in the vitamin D arm (Median: 33 ng/mL), compared to the placebo arm (Median: 19 ng/mL), and the increase persisted with the duration of the treatment (42 ng/mL vs. 22 ng/mL after 3 years treatment). Conversely, PTH decreased in the vitamin D arm as compared to placebo. No significant effect was observed between arms as regards serum hs-CRP concentrations. The multivariate random effects model for change in time of 25OHD is illustrated in Table 4.

**Figure 2:**
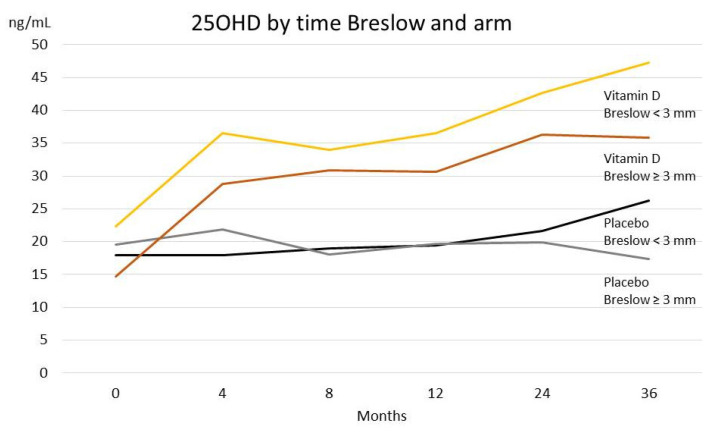
Difference in 25OHD increase by Breslow thickness.

Importantly, we observed a difference in 25OHD increase by Breslow thickness. While patients with Breslow thickness below 3 mm had a doubling of 25OHD serum levels from baseline after 12 months, patients with greater thickness experienced a much lower increases in 25OHD by time (Figure 2, Table 5).

**Figure 3:**
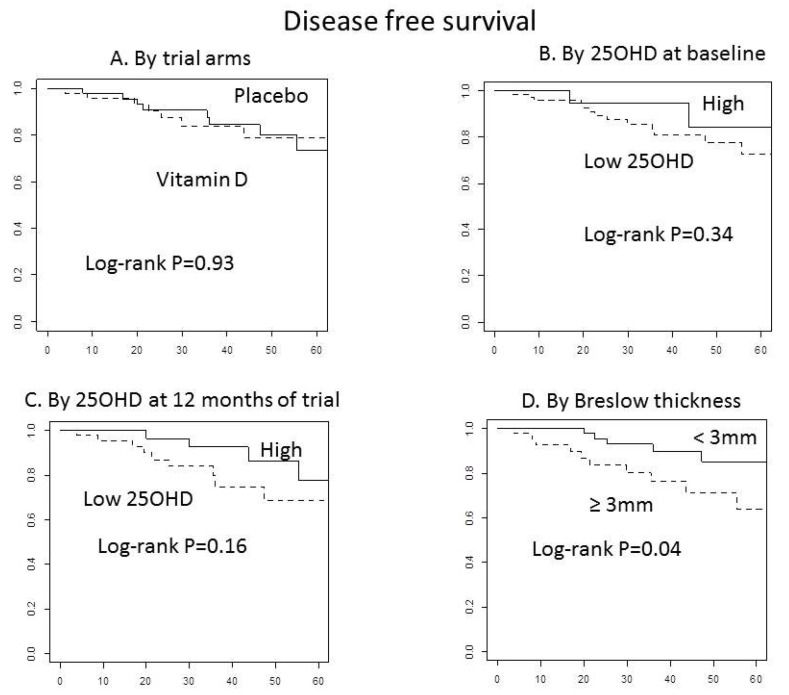
Diseas-free survival (Kaplan–Meier curve). (**A**) By trial arms, (**B**) By 25OHD at baseline, (**C**) By 25OHD at 12 months of trial, (**D**) By Breslow thickness.

**Figure 4:**
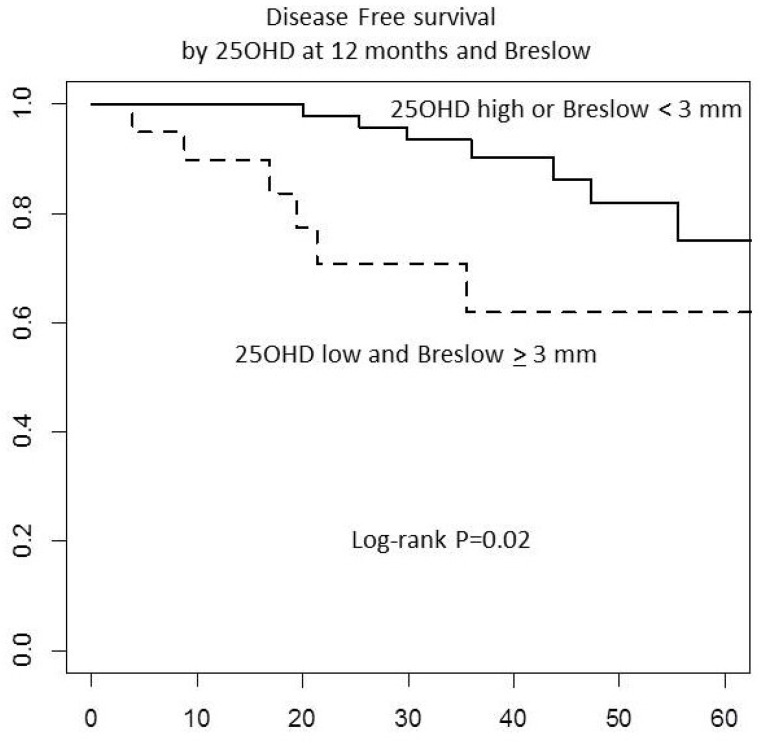
Disease-free survival by 25OHD levels at 12 months and Breslow score. The association of 25OHD levels with relapse was assessed considering 25OHD concentrations at baseline and at 12 months, after one year of supplementation or placebo.

After a median follow-up of 3 years, no difference by treatment in disease-free survival was observed (Figure 3A). Investigating the association of 25OHD with disease-free survival (Figure 3B,C), we found a trend for an inverse association of 25OHD with relapse after 12 month supplementation as compared to placebo (Figure 3C, *p* = 0.16). Noteworthy, Breslow thickness categorized as <3 and ≥3 mm was associated with disease-free survival (Figure 3D; *p* = 0.04). After adjusting for age, arm, and 25OHD levels at 12 months, the HR for participants with Breslow score <3 was 0.26 (95% CI: 0.08–0.90, *p* = 0.033) as compared to the HR for those with Breslow score ≥3. After 12 months of intervention, patients with both risk conditions (Breslow score ≥3 mm and low 25OHD levels), presented significantly lower disease-free survival (Figure 4; *p* = 0.02), compared to patients with any other condition (patients with 25OHD high levels and high Breslow score, or 25OHD high levels and low Breslow score, or 25OHD low levels and Breslow score <3). The association was confirmed in a multivariate Cox proportional hazard model, adjusting for age and treatment arms. The HR for recurrence was 4.81, (95% CI: 1.44–16.09; *p* = 0.011; Table 6) for patients with Breslow score ≥3 mm and low levels of 25OHD. When adjusting for ulceration or mitotic rate, the results did not change (Table S2). No interactions of hs-CRP, LDH, or NLR with prognosis were observed.

Overall, no severe adverse events were observed. Only 7% of the participants encountered grade 1–2 adverse events, while no grade 3–4 events were reported. Neither of the events were linked to 25OHD serum levels, nor were they associated with treatment allocation (*p* = 0.24).

All vitamin D-related polymorphisms (VDR, GC gene, and cytochrome P450 enzyme genes) were in Hardy–Weinberg equilibrium; SNP refs, minor allele frequencies (MAF), and genotype frequencies are illustrated in Table S3. VDR BsmI was found to be significantly associated with tumor stage. Carriers of the B allele (BB/Bb) had a lower risk of having a stage T3b–T4 tumor compared to bb genotype carriers (OR = 0.21; 95% CI: 0.07–0.69; *p* = 0.010) in a multivariable logistic model, adjusting for 25OHD.

The three SNPs of the gene coding for the main transporter of Vitamin D correlated with baseline 25OHD levels. The homozygote major allele genotypes had median 25OHD levels around 22 ng/mL, compared to the complementary genotype carriers that had median levels around 15–17 ng/mL (Table S4). Nevertheless, the genotype did not interact with treatment effects. No other SNP was associated with the circulating levels of 25OHD nor with treatment response and prognosis.

## 4. Discussion

Surgery is the main therapeutic option in cases with localized disease, and major prognostic factors are Breslow thickness, mitotic index, and ulceration. No adjuvant treatment is considered after excision of stage II melanoma. To our knowledge, this is the first study reporting results from a randomized, placebo-controlled trial investigating the effects of vitamin D supplementation on outcome in melanoma patients. Our study demonstrates an interaction between Breslow thickness and circulating levels of 25OHD. Despite taking 100,000 IU/every 50 days, participants who had a Breslow thickness ≥3 mm at diagnosis tended to experience a lower increase in 25OHD and were more prone to relapse in the future, as compared to participants with a low Breslow score at diagnosis. The low efficacy of vitamin D supplementation in patients with Breslow thickness ≥3 mm at diagnosis, could be related to a decreased expression rate of vitamin D-related proteins associated with cancer development and control, as well as other vitamin D-triggered changes in RNA expression of vitamin D-targeted genes contributing to vitamin D status [19]. Contrarily, an increase in serum 25OHD does not per se guarantee a high score of the personal vitamin D response index. Albeit the effort to extend accrual to other centers, we encountered lower accrual rates than expected, and the study was admittedly underpowered to meet the original primary end-point hypothesis criteria to prove any difference in disease-free survival between vitamin the D supplementation and placebo arms. Thus, in this cohort study, we investigated the associations of 25OHD changes and VDR polymorphisms with prognostic factors at baseline and disease-free survival. Another trial, the ViDMe phase III trial, adopting a dose of 100,000 IU/month or placebo for stage IB–III melanoma patients, is ongoing [20] and aims to confirm the hypothesis that vitamin D supplementation after tumor removal has a protective effect on recurrence of malignant melanoma; however, no results have been published to date.

Amongst factors that may contribute to a worse prognosis, chronic inflammation has emerged as an important player in health and disease outcome, and several recent findings point to a role also in skin melanoma outcome. CRP could play a role as a prognostic serological biomarker in early-stage melanoma patients [21]. A prospective study designed to identify factors that influence melanoma risk and clinical outcome was conducted at a single cancer center. They found that elevated CRP levels were associated with poorer overall survival and melanoma-specific survival [22]. In our randomized trial, having repeated measurements of CRP, we did not find any evidence of an association between hs-CRP and prognosis, 25OHD levels, or Breslow thickness. This result is partially in line with the study by Fang and co-authors, as CRP was not independently associated with risk of poorer prognosis [14], when including other risk modifiers. However, it is still unclear whether circulating CRP concentrations play a causal role in malignancy, because CRP is a nonspecific marker of many diseases [15], having either pro- or anti-inflammatory properties in relation to the context in which it is acting. Additional markers of inflammation have been investigated, and evidence from epidemiological studies points to a link between lower NLR and longer melanoma progression-free survival and overall survival [23,24]. A meta-analysis showed evidence of an increased risk of microscopic metastatic melanoma in the sentinel lymph node in the presence of higher NLR [25]. The immune-modulating ability of vitamin D could offer indications for novel applications in cancer patients receiving immunotherapy, to reinforce the anti-tumoral response, and to prevent and/or limit the onset of immune-related adverse events. It is worth mentioning that the timing of vitamin D supplementation in melanoma cancer prevention may play an important role. Vitamin D has been shown to prevent skin redness, and an experimental study showed that participants with higher serum vitamin D3 levels had increased skin expression of the anti-inflammatory mediator arginase-1 after treatment and a sustained reduction in skin redness that correlated with the expression of genes related to skin barrier repair [4]. Thus, it would be important to achieve sufficient vitamin D levels early on to prevent melanoma.

VDR expression has been associated with melanoma malignancy [26]. We found a suggestive inverse association of BsmI with melanoma stage, the B allele being associated with a lower stage as compared to the b allele. The VDR SNP BsmI is an intronic transition and does not alter the amino acid sequence of the translated protein. However, in Caucasians, this SNP is in strong linkage disequilibrium with a poly(A) microsatellite repeat in the 3′ region that influences VDR messenger RNA stability [27] and may affect local VDR protein levels [28]. A population-based study involving a cohort of 3566 primary melanoma patients [29] found that the B allele was statistically significantly associated with longer melanoma-specific survival outcomes, but not with Breslow thickness, ulceration, or mitosis. Although the relatively small number of studies on the association between melanoma and NMSC with Bsm1 polymorphism makes it difficult to draw a firm conclusion, our results are in agreement with findings from previous meta-analyses [30,31] suggesting a protective effect of the B allele on skin cancer risk and prognosis, likewise confirmed for other cancer types.

## 5. Conclusions

Our results support the hypothesis that vitamin D has a role in cutaneous melanoma prognosis. Vitamin D supplementation at 100,000 IU/every 50 days for three years is safe and well tolerated in patients with grade II melanoma cancer. Regardless the pronounced increase in 25OHD levels in the active arm, participants who had a Breslow thickness ≥3 mm at diagnosis experienced a lower increase in 25OHD levels and were more prone to relapse in the future, as compared to participants with a low Breslow score at diagnosis. Despite the evidence of a role of 25OHD in melanoma prognosis, larger prospective trials for patients with stage II melanoma cancer are needed to confirm a favorable long-term preventive effect of vitamin D supplementation on melanoma recurrence and survival. Vitamin D supplementation may represent a good prevention option for the low-risk group of melanoma patients, while the higher risk group might better benefit from a combination of vitamin D with an immunomodulatory agent, although this approach needs validation.

## Figures and Tables

**Table 1:** Descriptive baseline characteristics of the included patients.

	Groups	All	Placebo (*n* = 52)	Vitamin D (*n* = 52)	*p*-Value
Gender, *n* (%)	Males	59 (56.7)	27 (51.9)	32 (61.5)	0.322
	Female	45 (43.3)	25 (48.1)	20 (38.5)	
Staging, *n* (%)	stage IIa T2b	19 (18.3)	9 (17.3)	10 (19.2)	0.988
	stage IIa T3a	41 (39.4)	20 (38.5)	21 (40.4)	
	stage IIb T3b	28 (26.9)	14 (26.9)	14 (26.9)	
	stage IIb T4a	9 (8.7)	5 (9.6)	4 (7.7)	
	stage IIc T4b	7 (6.7)	4 (7.7)	3 (5.8)	
Ulceration, *n* (%)	No	46 (44.2)	23 (44.2)	23 (44.2)	0.839
	Yes	55 (52.9)	28 (53.8)	27 (51.9)	
	Missing	3 (2.9)	1 (1.9)	2 (3.8)	
Mitotic rates, *n* (%)	<2	26 (25)	13 (25)	13 (25)	0.494
	≥2	71 (68.3)	34 (65.4)	37 (71.2)	
	Missing	7 (6.7)	5 (9.6)	2 (3.8)	
25OHD	Medium/high	21 (20.2)	9 (17.3)	12 (23.1)	
*n* (%)	Low *	83 (79.8)	43 (82.7)	40 (76.9)	
	n.	Median (IQR)	Median (IQR)	Median (IQR)	
Age, years	104	50.5 (41.5, 59)	50 (42, 56.5)	52 (40.5, 62.5)	0.688
Breslow, mm.	104	2.62 (2.13, 3.8)	2.62 (2.17, 3.8)	2.65 (2.1, 3.7)	0.861
25OHD, ng/ml	104	18.1 (12.7, 24.3)	18.2 (13.4, 24.3)	18.1 (12, 23.9)	0.768
LDH	85	283 (201, 356)	280 (186, 350)	285 (201, 367)	0.389
NLR	87	1.31 (1.00, 1.8)	1.54 (1.07, 1.83)	1.22 (0.93, 1.69)	0.138

IQR: Interquartile range. * Low vitamin D: 25OHD below 20 ng/mL from December to July and below 30 ng/mL from August to November. LDH = serum lactate dehydrogenase; NLR = neutrophil/lymphocyte ratio.

**Table 2:** Patients with melanoma Stage IIa versus IIb/IIc by 25OHD and VDR BsmI at baseline.

	Stage IIa	Stage IIb/IIc		
**25OHD (ng/mL)**	T2 or T3a *n* = 60	T4 or T3b *n* = 44	**Total**	*p*-Values *
Medium/high	14 (23%)	7 (16%)	21	0.35
Low	46 (77%)	37 (84%)	83	
**BsmI**	*n* = 58	*n* = 43		
bb/bB	40 (69%)	39 (91%)	79	0.009
BB	18 (31%)	4 (9%)	22	

* Chi-square test.

**Table 3:** Median and interquartile ranges of serum 25OHD, PTH, and hs-CRP according to treatment arm and time interval.

	Number of Subjects		25 OHD (ng/mL)		PTH (pg/mL)		hs-CRP (mg/dL)	
Time-Point	Placebo	Vitamin D	Placebo	Vitamin D	Placebo	Vitamin D	Placebo	Vitamin D
Baseline	52	52	18.15 (13.4–24.2)	18.0 (12.0–23.9)	19.8 (15.2–28.9)	21.0 (15.0–25.4)	0.12 (0.06–0.20)	0.14 (0.06–0.25)
4 months	50	42	19.05 (13.0–25.9)	32.9 (25.9–38.4)	22.1 (15.9–30.3)	19.3 (13.8–26.0)	0.12 (0.07–0.26)	0.12 (0.05–0.22)
8 months	50	42	18.7 (13.0–22.9)	33.4 (25.1–37.1)	20.2 (16.2–28.1)	18.6 (14.0–24.6)	0.10 (0.05–0.19)	0.14 (0.05–0.34)
12 months	44	44	19.4 (14.3–25.0)	33.8 (28.0–40.0)	22.2 (17.2–29.6)	16.5 (13.7–24.4)	0.12 (0.07–0.20)	0.12 (0.06–0.25)
24 months	37	32	21.1 (15.0–26.0)	39.2 (31.6–46.5)	24.0 (18.0–26.9)	19.2 (14.6–25.8)	0.12 (0.08–0.25)	0.10 (0.05–0.20)
36 months	25	22	22.5 (15.9–28.8)	41.9 (31.8–47.3)	26.0 (19.0–31.2)	19.7 (17.8–26.2)	0.10 (0.05–0.15)	0.06 (0.05–0.25)
48 months	9	9	21.4 (14.3–25.7)	31.2 (24.3–35.5)	22.9 (17.8–27.2)	23.4 (22.5–30.4)	0.10 (0.04–0.12)	0.10 (0.03–0.23)
60 months	5	6	17.8 (14.3–24.1)	22.4 (20.3–27.1)	32.0 (30.7–32.4)	25.6 (25.5–26.0)	0.12 (0.03–0.13)	0.12 (0.05–0.58)

**Table 4:** Multivariate random effects model for estimates of change in time of 25OHD.

		Change in 25OHD	Std Error	*p*-Values
Intercept		20.91	2.69	<0.0001
Baseline 25OHD		−0.51	0.09	<0.0001
Season	Late summer/autumn	3.96	1.36	0.004
	Winter	−3.75	1.31	0.005
	Spring/early summer	0.00		
Arms	Placebo	0.00		
	Vitamin D	12.01	2.39	<0.0001
Placebo by time	4	0.00		
	8	−1.16	2.05	0.572
	12	−1.69	2.15	0.431
	24	−0.39	2.22	0.859
	36	4.06	2.51	0.107
Vitamin D by time	4	0.00		
	8	3.30	2.27	0.147
	12	2.74	2.25	0.223
	24	6.43	2.43	0.009
	36	9.61	2.71	0.001
Breslow	<3 mm	2.83	1.48	0.058
	≥3 mm	0.00		

**Table 5:** Least-square means (LSM) of 25OHD by arms, time, and Breslow thickness from the multivariable random effects model adjusted for season and baseline level.

	Vitamin D				Placebo		
Breslow	Months	LSM 25OHD (ng/mL)	Standard Error	*p*-Values	LSM 25OHD (ng/mL)	Standard Error	*p*-Values
<3 mm	4	14.23	2.65	<0.0001	−0.77	1.62	0.64
	8	18.79	2.68	<0.0001	−0.29	1.62	0.86
	12	16.76	2.69	<0.0001	0.21	1.66	0.90
	24	20.28	2.91	<0.0001	1.57	1.70	0.36
	36	30.50	3.78	<0.0001	8.86	2.12	<0.0001
≥3 mm	4	10.75	3.54	0.003	2.71	1.82	0.14
	8	11.31	3.34	0.001	−0.08	1.98	0.97
	12	12.75	3.08	<0.0001	−1.92	2.19	0.38
	24	18.33	3.70	<0.0001	−0.67	2.37	0.78
	36	14.51	3.79	0.0002	−1.51	2.70	0.58

**Table 6:** Multivariate Cox proportional hazard model for recurrence.

		At Baseline				At 12 Months			
		HR	Low 95% CI	High 95% CI	*p*-Value	HR	Low 95% CI	High 95% CI	*p*-Value
Age		1.03	0.99	1.07	0.21	1.04	0.99	1.09	0.104
Arm	Placebo vs. Vitamin D	0.98	0.34	2.84	0.97	0.82	0.22	3.14	0.774
25OHD	High vs low *	0.58	0.12	2.68	0.48	3.15	0.74	13.43	0.121
Breslow	<3 mm vs. ≥3 mm	0.35	0.12	1.03	0.06	0.26	0.08	0.90	0.033
Age		1.03	0.99	1.07	0.21	1.04	0.99	1.09	0.094
Arm	Placebo vs. Vitamin D	0.99	0.36	2.76	0.98	0.81	0.26	2.57	0.723
25OHD Breslow combined score	25OHD low and Breslow ≥3 vs. (25OHD high or Breslow <3) **	2.36	0.85	6.51	0.10	4.81	1.44	16.09	0.011

* Low vitamin D: 25OHD below 20 ng/mL from December to July and below 30 ng/mL from August to November. ** Specifically, patients with 25OHD high levels and high Breslow score, or 25OHD high levels and low Breslow score, or 25OHD low levels and Breslow score <3.

## Data Availability

The data underlying this article may be shared upon reasonable request to the coordinating center of the trial. Contact Sara Gandini, Experimental Oncology, IEO, European Institute of Oncology IRCCS, Milan.

## Supplementary Materials

The following are available online at https://www.mdpi.com/article/10.3390/nu13061931/s1, Table S1: Centers involved in the study and number of patients recruited, Table S2: Multivariate cox proportional hazard model for disease-free survival, Table S3: Vitamin D polymorphisms, minor allele frequency (MAF), and genotype frequencies, Table S4: GC polymorphisms and 25OHD serum levels (ng/mL).
