# Supplementary material for: Vitamin D Supplementation and Disease-Free Survival in Stage II Melanoma: A Randomized Placebo Controlled Trial
Source: Nutrients. 2021 Jun 4;13(6):1931. doi: 10.3390/nu13061931 (PMC8226808; doi:10.3390/nu13061931)
Supplement: Supplementary file 1 [file nutrients-13-01931-s001.zip › nutrients-1230158-supplementary.pdf]

Supplemental Table S1. Centers involved in the study and number of patients recruited.

CENTERS	TOTAL N, %
Istituto Europeo di Oncologia - Milano	58 (56%)
Azienda Ospedaliera Papa Giovanni XXIII - Bergamo	23 (22%)
A. O. U. "S. Maria della Misericordia" - Udine	13 (13%)
I.R.C.C.S. A.O.U. San Martino – IST di Genova	8 (7%)
P.O.S. Antonio Abate – A.S.P. Trapani - Erice	1 (1%)
P. O. Antonio Perrino – Brindisi	1 (1%)
**TOTALE**	104 (100%)

Supplemental Table S2. Multivariate Cox proportional hazard model for Disease free Survival.

	HR	Low 95%CI	Up 95%CI	P-value
Age	1.04	0.98	1.09	0.19
Arm Placebo vs Vitamin D	0.73	0.22	2.46	0.62
VD low and Breslow≥3 vs (VD high or Breslow<3)	5.19	1.53	17.57	0.01
Ulceration yes vs no	1.10	0.36	3.39	0.87
Age	1.03	0.98	1.08	0.25
Arm Placebo vs Vitamin D	0.83	0.25	2.71	0.75
VD low and Breslow≥3 vs (VD high or Breslow<3)	6.03	1.80	20.26	<0.001
N. mitosis>2 vs ≤2	3.87	0.46	32.53	0.21

Supplemental Table S3. Vitamin D polymorphisms, minor allele frequency (MAF).

Ref Seq	MAF S480	MAF HapMap CEU	Genotype frequencies		
rs1544410; VDR BsmI, C/T*	T (0.46)	0.44	30 (CC)	49 (CT)	22 (TT)
rs731236; VDR TaqI, A/G	G (0.45)	0.44	32 (AA)	47 (AG)	22 (GG)
rs7975232; VDR ApaI, A/C	C (0.41)	0.43	33 (AA)	55 (AC)	13 (CC)
rs2228570; VDR FokI, A/G	A (0.40)	0.41	37 (GG)	48 (GA)	16 (AA)
rs4588; G/T	T (0.27)	0.27	50 (GG)	43 (GT)	8 (TT)
rs7041; A/C	A (0.41)	0.42	33 (CC)	46 (CA)	22 (AA)
rs2282679; G/T	G (0.26)	0.26	51 (TT))	42 (TG)	8 (GG)
rs6013897; CYP24A1, T/A	A (0.24)	0.26	55 (TT)	40 (TA)	6 (AA)
rs10877012; CYP27B1, G/T	T (0.27)	0.33	56 (GG)	35 (GT)	10 (TT)
rs10741657; CYP2R1, A/G	A (0.28)	0.38	59 (GG)	33 (GA)	9 (AA)

*C=b and T=B according to restriction fragmentation genotyping with BsmI.

Supplemental Table S4. GC polymorphisms and 25OHD serum levels (ng/mL).

	25OHD Median (IQ range) Major allele homozygote genotype	25OHD Median (IQ range) Alternative genotypes
rs2282679 TT (n=51) vs rs2282679 GG/GT (n=50)	22.0 (15.4-25.9)	15.0 (11.9-20.0)
rs4588 GG (n=50) vs rs4588 GT/TT (n=51)	21.8 (15.2-25.9)	15.0 (12.0-21.0)
rs7041 CC (n=33) vs rs7041 AA/AC (n=68)	21.6 (14.4-25.4)	16.9 (12.1-23.4)
Major allele haplotype (n=33) vs no major allele haplotypes (n=50)	21.6 (14.4- 25.4)	15.0 (11.9-20.0)
